# eDGAR: a database of Disease-Gene Associations with annotated Relationships among genes

**DOI:** 10.1186/s12864-017-3911-3

**Published:** 2017-08-11

**Authors:** Giulia Babbi, Pier Luigi Martelli, Giuseppe Profiti, Samuele Bovo, Castrense Savojardo, Rita Casadio

**Affiliations:** 1 Biocomputing Group, BiGeA, University of Bologna, Bologna, Italy; 2 Interdepartmental Center «Giorgio Prodi» for Cancer Research, University of Bologna, Bologna, Italy

**Keywords:** Gene/disease relationship, Protein-protein interaction, Protein functional annotation, Functional enrichment

## Abstract

**Background:**

Genetic investigations, boosted by modern sequencing techniques, allow dissecting the genetic component of different phenotypic traits. These efforts result in the compilation of lists of genes related to diseases and show that an increasing number of diseases is associated with multiple genes. Investigating functional relations among genes associated with the same disease contributes to highlighting molecular mechanisms of the pathogenesis.

**Results:**

We present eDGAR, a database collecting and organizing the data on gene/disease associations as derived from OMIM, Humsavar and ClinVar. For each disease-associated gene, eDGAR collects information on its annotation. Specifically, for lists of genes, eDGAR provides information on: i) interactions retrieved from PDB, BIOGRID and STRING; ii) co-occurrence in stable and functional structural complexes; iii) shared Gene Ontology annotations; iv) shared KEGG and REACTOME pathways; v) enriched functional annotations computed with NET-GE; vi) regulatory interactions derived from TRRUST; vii) localization on chromosomes and/or co-localisation in neighboring loci. The present release of eDGAR includes 2672 diseases, related to 3658 different genes, for a total number of 5729 gene-disease associations. 71% of the genes are linked to 621 multigenic diseases and eDGAR highlights their common GO terms, KEGG/REACTOME pathways, physical and regulatory interactions. eDGAR includes a network based enrichment method for detecting statistically significant functional terms associated to groups of genes.

**Conclusions:**

eDGAR offers a resource to analyze disease-gene associations. In multigenic diseases genes can share physical interactions and/or co-occurrence in the same functional processes. eDGAR is freely available at: edgar.biocomp.unibo.it

**Electronic supplementary material:**

The online version of this article (doi:10.1186/s12864-017-3911-3) contains supplementary material, which is available to authorized users.

## Background

The advent of fast and relatively costless techniques for genome screening boosts the research of genetic determinants of human phenotypes, with a specific focus on diseases [1]. By this, lists of genes involved in several diseases/phenotypes are available. One of the most comprehensive database of curated associations between human Mendelian disorders and genes is OMIM [2], collecting 4510 phenotypes with known molecular basis (release of May 2016). Updated resources of associations between variations and diseases are stored in the NCBI-curated ClinVar [3], the UniProt curated Humsavar list [4], and the commercial version of HGMD [5]. Integrative datasets, such as DisGeNet [6] and MalaCards [7] collect lists of gene-disease associations from different sources. MalaCards includes text mining of the scientific literature, gene annotations in terms of shared GO terms and associated pathways. DisGeNet integrates data of disease-associated genes and their variants. Furthermore, a database collecting data on digenic diseases (related to concomitant defects in pairs of genes) is available (DIDA, [8]) and reports the relationships between pairs of genes involved in 44 diseases.

As data accumulate, it emerges that an increasing number of diseases is associated with several genes. Independent or concomitant alterations in sequence or in expression of sets of genes are associated with the insurgence of genetically heterogeneous and polygenic diseases, respectively [9, 10]. The scenario is even more complicated when different environmental and life-style related factors have strong influence on the insurgence and severity of the pathology [11]. The complex nature of the association between genes and diseases is one of the major challenges of Precision Medicine programs [12].

Dissecting the molecular mechanisms at the basis of the association between genotype and phenotype requires a deep investigation of the features shared among genes (or proteins) co-involved in the same disease. Indeed, by analyzing molecular features and functional interactions, important biological processes and pathways implicated in the disease can emerge and other genes possibly involved in interaction networks can be discovered [13, 14].

This work describes eDGAR, a database of gene-disease associations, supplemented with the annotations of intergenic relationships in heterogeneous and polygenic diseases. We merged, without redundancy, data from OMIM [2], ClinVar [3], and Humsavar [4]. Disease nomenclature derives from OMIM. OMIM phenotype entries are classified according to the OMIM Phenotypic Series, which cluster different entries related to identical or highly similar diseases associated with different genes. As compared to the above mentioned databases, our focus is on specific structural and functional annotations of the genes. For each gene, the database reports the cytogenetic location, links to the Ensembl [15], SwissProt [4] and PDB entries [16], Gene Ontology (GO) [17] annotations and to the KEGG and REACTOME pathways, when available. For sets of genes involved in the same disease, the database collects from publicly available databases different types of relationships: physical interactions, co-occurrence in protein complexes, regulatory interactions, shared functions and pathways, and co-localization in neighboring cytogenetic loci. A network - based approach (NET-GE [18, 19]) provides statistical enrichment to functional terms. Information is organized in a relational database and an interface allows customized data search and retrieval.

The database is freely available at edgar.biocomp.unibo.it.

## Construction and content

### Data sources of associations between genes and diseases

In order to collect a comprehensive resource of associations among genes and diseases we integrated data from OMIM (May 2016 release) [2], ClinVar (May 2016 release) [3] and Humsavar (June 2016 release) [4]. The primary accessions for genes are HGNC codes [20], while OMIM identifiers are adopted to identify phenotypes. 2839 OMIM phenotype codes corresponding to identical or similar diseases, characterized by genetic heterogeneity, have been clustered into 357 phenotypic series, as defined by OMIM. Synonymic or alternative gene names were reduced to the HGNC gene primary codes, as reported in HGNC (June 2016 release).

On the overall, 5337, 4358 and 3365 gene-disease associations were collected from OMIM, ClinVar and Humsavar, respectively, by retaining only associations with unambiguous identification codes for both genes and diseases. After removing redundancy, the final dataset contains 5729 gene-disease associations, involving 3658 genes associated with 2672 diseases. These 2672 disease IDs correspond to 2315 OMIM IDs for phenotypes and 357 phenotypic series, or to 5154 when the 357 phenotypic series are brought back in 2839 OMIM IDs for phenotypes.

### Gene annotation

All genes have been associated with the corresponding Ensembl codes (June 2016 version) [15] with BioMart [21]. Cytogenetic locations on the GrCh38 version of the human genome were therefrom derived. Out of 3658, 30 genes encode for microRNAs and tRNAs. For the 3628 protein coding genes, links to the SwissProt and PDB databases were also retrieved: all genes are linked to at least one SwissProt entry (for a total of 3718 entries) and 1682 genes are linked to at least one PDB entry (for a total of 14,578 PDB entries).

Functional annotation based on Gene Ontology (GO) terms was retrieved from GOOSE, the Online SQL Environment for GO terms implemented in the AmiGO2 portal [22]. All three GO sub-ontologies (Molecular Function: MF; Biological Process: BP; Cellular Component: CC) were considered. Given a GO term, the ancestor terms in the directed acyclic graph of GO (version 2.4) were retrieved by considering the relations “is a subtype of” and “part of”. The information content (IC) was computed for each GO term, adopting standard methods [23], with the following equation:

1 $$ IC=-{log}_2\left(\frac{N_{GO}}{N_{root}}\right) $$

where *N*_*GO*_ is the number of human genes endowed with the particular GO term and *N*_*root*_ is the number of human genes annotated with all the terms of the considered subontology, as derived from GOOSE [22]. IC lower limit is zero; high IC values indicate that a small number of genes is annotated with a particular GO term in the human genome and therefore the annotation is highly informative.

Associations with KEGG (version 77.0) [24] and REACTOME (version 53) [25] pathways were extracted from SwissProt.

### Relationships among genes involved in the same disease

eDGAR integrates several information in order to annotate the possible relationships among protein coding genes related to the same polygenic or heterogeneous disease. The following features are considered:

- Protein-protein interactions, as derived from the multimeric structures deposited at the PDB (February 2016 release) [16], from STRING (version 10.0) [26] and from the experimental data available in BIOGRID (version 3.4) [27]. From the human STRING network, we retained only high confidence links (score ≥ 0.7) with annotated “action”. Physical and genetic interactions of BIOGRID are reported separately. For all the considered human interactomes, eDGAR reports both direct and indirect interactions involving one intermediate gene. In addition, we supplemented data on interactions with selected annotations from manually curated features from SwissProt, including links to the PDB and the literature.
- Interactions in stable and functional complexes reported in the following resources: CORUM, listing 2837 mammalian complexes involving 3198 protein chains (16% of the human protein-coding genes) [28], the soluble complex census, listing 622 complexes involving 3006 protein chains [29]. This last resource is referred in the following as CENSUS.
- Functional GO terms and KEGG/REACTOME pathways shared by at least two genes.
- Functional GO terms and KEGG/REACTOME pathways retrieved with NET-GE [18, 19], a network based tool that performs the statistically-validated enrichment analysis of sets of human genes by exploiting the human STRING interactome; a significance of 5% was considered when retrieving statistically enriched terms on the basis of the Bonferroni-corrected *p*-values computed with NET-GE;
- Regulatory interactions derived from TRRUST [30], a curated database of interactions among 748 human transcription factors (TF) and 1975 non-TF targets. Given a set of genes associated with the same disease, eDGAR reports the presence of TF/target pairs and of groups of genes co-regulated by the same TF (belonging or not to the set);
- Co-localization in neighboring loci on the same chromosome: we highlighted genes located in the same cytogenetic band or in the tandem repeat regions listed in the DGD database [31]. DGD collects 945 groups consisting of 3543 genes in humans, likely deriving from duplications of ancestor genes.

### Database structure and visualization

The database is implemented with PostgreSQL [32], an open source relational database system. Data stored in the database are retrieved using custom Python programs, while the output of the analysis is visualized in HTML pages using modern technologies like JavaScript. In particular, networks are encoded in JSON format and visualized using the JavaScript library D3.js [33]. We adopted a well known plug-in for jQuery called DataTables [34] for table visualizations, allowing the user to sort tables by columns and text-search inside each table.

## Results and discussion

### Statistics of the database content

**Fig. 1:**
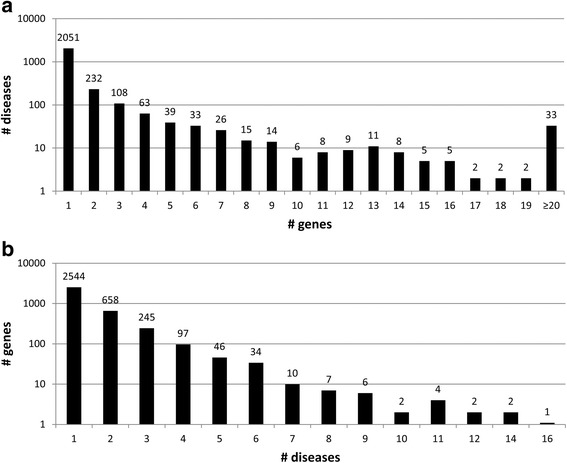
Distribution of gene-disease associations. The Y-axis scale is logarithmic. **a** Number (#) of genes associated with diseases. 2672 diseases are distributed with respect to the number of associated genes. 2051 diseases are monogenic; 621 diseases are associated with multiple genes (from 2 to 69). **b** Number (#) of diseases associated to genes. 3658 genes are distributed with respect to the number of associated diseases. 2544 genes are associated with a single disease; 1114 genes are associated with multiple diseases (from 2 to 16)

The present release of eDGAR collects 5729 associations between 2672 diseases and 3658 different genes. Figure 1a plots the distribution of the number of genes associated with the same disease, which ranges from one (in 2051 monogenic diseases) to 69 (in the case of the “Retinitis pigmentosa” phenotypic series, OMIM: PS268000). The 621 diseases associated with multiple genes comprise both heterogeneous and polygenic diseases. On the overall, they account for 3678 associations with 2600 genes, 2576 of which code for proteins.

The database also shows a high level of pleiotropy (association of a single gene to several diseases) as shown in Fig. 1b. The most pleiotropic gene is FGFR3 that codes for the fibroblast growth factor receptor 3 and is associated with 16 different diseases.

### Statistics of gene annotation

Table 1 lists major annotations of the 3658 genes related to diseases. All but 30 genes are coding for proteins reported in SwissProt; for 46.4% of them, structural information is available in PDB. Membrane proteins, transcription factors and enzymes account for 52%, 7% and 31%, respectively. Almost all the protein-coding genes are functionally annotated: the fraction of genes endowed with GO terms ranges from 94.2% to 98.6%, depending on the sub-ontology (Molecular Function (MF), Biological Process (BP) and Cellular Component (CC)). A smaller percentage of genes are associated with KEGG and REACTOME pathways (56.7% and 62.8%, respectively).

**Table 1:** Gene annotation in eDGAR

	All diseases		Diseases associated with multiple genes	
	# genes^a^	# associated diseases^b^	# genes^a^	# associated diseases^b^
Total number	3658	2672	2600	621
Protein coding genes	3628 (100%)	2655 (100%)	2576 (100%)	619 (100%)
with PDB entry	1682 (46.4%)	1625 (61.2%)	1176 (45.7%)	512 (82.7%)
Membrane proteins	1891 (52.1%)	1644 (61.9%)	1364 (53.0%)	517 (83.5%)
Enzymes (with E.C number)	1112 (30.7%)	1045 (39.4%)	688 (26.7%)	363 (58.6%)
Reported in TRRUST (as TF)	253 (7.0%)	358 (13.5%)	179 (6.9%)	157 (25.4%)
Reported in TRRUST (as target)	783 (21.6%)	969 (36.5%)	570 (22.1%)	405 (65.4%)
Annotated with GO MF	3419 (94.2%)	2575 (97.0%)	2419 (93.9%)	617 (99.7%)
Annotated with GO BP	3538 (97.5%)	2619 (98.6%)	2514 (97.6%)	618 (99.8%)
Annotated with GO CC	3576 (98.6%)	2644 (99.6%)	2533 (98.3%)	618 (99.8%)
Associated with KEGG pathways	2057 (56.7%)	1868 (70.4%)	1430 (55.5%)	549 (88.7%)
Associated with REACTOME	2278 (62.8%)	2007 (75.6%)	1595 (61.9%)	563 (91.0%)
With physical BIOGRID interactions	3307 (91.3%)	2502 (94.2%)	2346 (91.2%)	609 (98.4%)
With genetic BIOGRID interactions	351 (9.7%)	472 (17.8%)	259 (10.1%)	247 (39.9%)
With STRING interactions	2992 (82.5%)	2341 (88.2%)	2146 (83.3%)	609 (98.4%)
Part of CORUM complexes	714 (19.7%)	706 (26.6%)	558 (21.7%)	340 (54.9%)
Part of CENSUS complexes	696 (19.2%)	689 (26.0%)	501 (19.4%)	296 (47.8%)
In tandem repeats	381 (10.5%)	448 (16.9%)	280 (10.9%)	234 (37.8%)

^a^Percentages are computed with respect to the number of protein coding genes

^b^Percentages are computed with respect to the number of diseases associated with protein coding genes

When considering human interactomes, 91.3% and 9.7% of the genes are present in BIOGRID with physical and genetic interactions, respectively; for 82.5% of the genes, STRING reports high confidence interactions (score ≥ 0.7). Some 20% of the genes encode for protein chains involved in functional complexes, as described in the CORUM and CENSUS collections. TRRUST lists some 1036 genes as part of the human regulatory network, of which 253 code for TFs and 783 are non-TF targets.

The level of annotation of the 2576 protein coding genes involved in heterogeneous or polygenic diseases is similar to that of all the genes collected in eDGAR.

### Relations among genes associated with the same disease

eDGAR lists the relations among different genes associated with the same multigenic disease (statistics is in Table 2). 21.9% of diseases involve at least one pair of genes located in the same cytogenetic band and in 8.2% of the cases, genes are tandem repeats originated by duplications. These genes are likely to undergo the same regulation mechanisms and to be coexpressed [33].

**Table 2:** Features shared by genes involved in the same heterogeneous or polygenic diseases

	# diseases	# pairwise relations	# protein coding genes
Total number	621	25,100	2576
With pairs of genes:			
In same cytogenetic band	136 (21.9%)	326 (1.3%)	335 (13.0%)
In tandem repeat	51 (8.2%)	58 (0.2%)	92 (3.6%)
In TF/target pairs	39 (6.3%)	81 (0.3%)	94 (3.6%)
Co-regulated by the same TF (not involved in the disease)	273 (44.0%)	2308 (9.2%)	626 (24.3%)
Sharing MF GO	586 (94.4%)	19,075 (76.0%)	2369 (92.0%)
Sharing BP GO	597 (96.1%)	22,948 (91.4%)	2502 (97.1%)
Sharing CC GO	604 (97.3%)	23,645 (94.2%)	2519 (97.8%)
Sharing KEGG pathway	349 (56.2%)	3129 (12.5%)	1074 (41.7%)
Sharing REACTOME pathway	474 (76.3%)	9806 (39.1%)	1554 (60.3%)
Interacting in PDB	96 (15.5%)	207 (0.8%)	199 (7.7%)
In the same CORUM complex	86 (13.8%)	469 (1.9%)	225 (8.7%)
In the same CENSUS complex	45 (7.2%)	166 (0.7%)	119 (4.6%)
Directly linked in STRING	291 (46.9%)	1535 (6.1%)	932 (36.2%)
Indirectly linked in STRING	115 (18.5%)	4355 (17.4%)	1346 (52.3%)
Directly linked in BIOGRID (physical interaction)	250 (40.3%)	944 (3.8%)	799 (31.0%)
Indirectly linked in BIOGRID (physical interaction)	160 (25.8%)	5228 (20.8%)	1607 (62.4%)
Directly linked in BIOGRID (genetic interaction)	9 (1.4%)	13 (0.1%)	19 (0.7%)
Indirectly linked in BIOGRID (genetic interaction)	25 (4.0%)	45 (0.2%)	62 (2.4%)

**Fig. 2:**
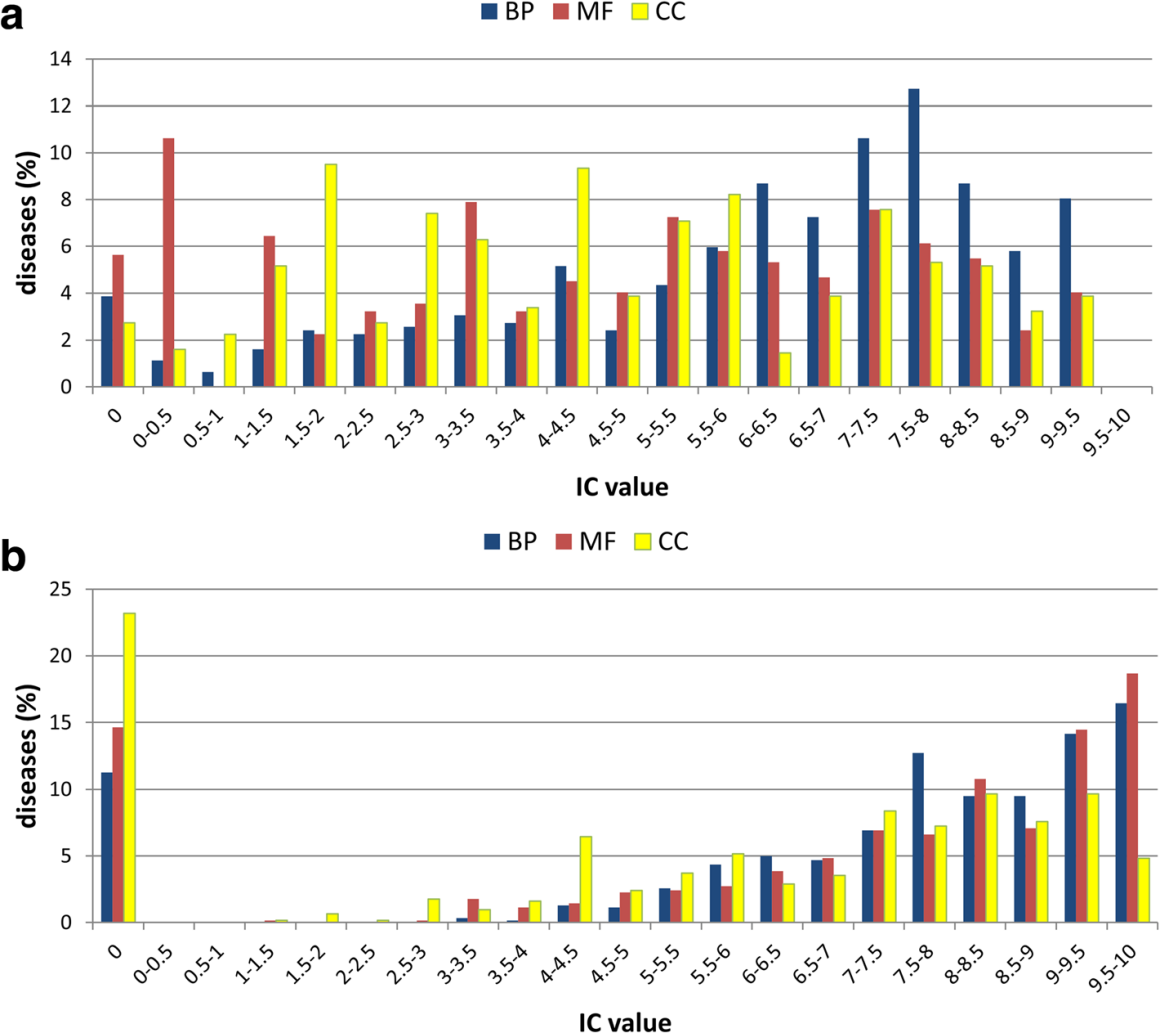
Distribution of best IC values of GO terms for genes involved in multigenic diseases. **a** GO terms shared by genes; **b** GO terms after enrichment with NET-GE. For each multigenic disease, IC values of gene-associated GO terms (of the three different roots) are evaluated (Eq. 1). In the figure, the highest IC for each disease is shown. The frequency is computed with respect to the total number of multigenic diseases (621). When IC = 0, genes associated with multigenic disease do not share or enrich GO terms (panel **a** and **b** respectively)

Many diseases involve at least one pair of genes directly linked in interactomes: 40.3% and 46.9%, considering BIOGRID or STRING networks, respectively. The rates increase to 66.1% and 65.4% when considering also indirect interactions involving one intermediate gene not associated with the disease. 6.3% of diseases involve pairs of genes in a Transcription Factor (TF)/target relationship and 44% involve genes co-regulated by the same TF (considering also TFs not directly associated with the disease). The large majority of diseases (from 94.4% to 97.3%, depending on the sub-ontology) is associated with at least one pair of genes sharing GO terms. More than 90% of all the possible pairs of genes involved in the same disease have common BP and CC terms; the percentage is somehow smaller (76%) for MF sub-ontology. The total number of GO annotations shared by pairs of genes for BP, MF and CC is 72,787 (unique terms: 4582), 13,113 (unique terms: 915) and 16,298 (unique terms: 656), respectively. Overall, these data confirm the notion that genes associated with the same disease share some level of functional similarity, a view previously suggested for a small number of multigenic diseases [14]. However, being GO terms organized in a directed acyclic graph for each root, the information conveyed by the shared annotations can be very different, going from very general to very specific terms. The information content (IC, see Eq. 1) is routinely associated with GO terms in order to evaluate their specificity with respect of the available annotation of all human genes. The IC values of our dataset range from 0 (corresponding to the root GO term) to 10 (corresponding to the most specific terms). The average IC values for MF, BP and CC shared terms are 5.8 ± 1.7, 5.9 ± 1.7, and 5.8 ± 1.9, respectively. For each disease, the specificity of the annotation is evaluated by extracting the best IC values among the GO terms shared by pairs of co-associated genes (Fig. 2a). For all the sub-ontologies, the best IC values are very spread, and it is evident that on average the most specific terms (highest IC values) belong to the BP sub-ontology: genes pairs sharing BP, MF and CC terms with IC ≥ 5 are present in 72%, 49% and 46% of the diseases, respectively (see Fig. 2a). When a different distribution based on a median is adopted, the pattern is very similar (Additional file 1: Fig. S1A). Genes involved in the same disease share also KEGG and REACTOME pathways (56.2% and 76.3%, respectively (Table 2)).

### NET-GE enrichment

In order to better highlight functions shared by groups of genes associated with the same disease, we adopt NET-GE [18, 19], our recently developed network based tool for functional enrichment. For each functional sets of GO terms and/or KEGG or REACTOME pathways, NET-GE builds a network containing all the human genes annotated with the terms (seeds) and including all the connecting genes (the reference human interactome is derived from STRING). Input genes are mapped into the pre-computed NET-GE networks and enrichment analysis is performed. Outputs are Bonferroni-corrected *p*-values, measuring the overrepresentation of each term in the input set. Due to its network-based nature, NET-GE can enrich terms not present in the list of annotations of the input set. Table 3 lists the results of NET-GE on the groups of genes associated with the same disease, considering a 5% significance. For the majority of diseases, NET-GE enriches GO terms of the three sub-ontologies and pathways of KEGG and REACTOME. BP is the sub-ontology type most frequently enriched. The total number of GO annotations enriched for heterogeneous and polygenic diseases is 17,029, 4851 and 3910 (Table 3, rightmost column), with average IC values 6.1 ± 1.8, 7.1 ± 2, and 6.4 ± 2 for BP, MF and CC respectively (Fig. 2b, reporting the distribution of the best IC values among the terms enriched for each disease; for a different distribution based on IC median values, see Additional file 1: Figure S1B).

**Table 3:** NET-GE functional enrichment of groups of genes involved in the same disease

	# diseases	# annotations
KEGG pathways	412 (66.3%)	2753
REACTOME pathways	488 (78.6%)	4130
GO MF terms	530 (85.3%)	4851
GO BP terms	551 (88.7%)	17,029
GO CC terms	477 (76.8%)	3910

### The user interface

eDGAR is publicly available as a web server at edgar.biocomp.unibo.it with browsing and search options. Browsing is performed with the “Main Table” page that contains all the collected associations between genes and diseases, along with the indication of source databases.

The Search engine allows to access the database with different identifiers: HGNC symbols and Ensembl identifiers for genes, UniProt accession for proteins, OMIM identifiers or disease names for phenotypes and phenotypic series. The user may also search with a set of genes and retrieve shared annotation features.

**Fig. 3:**
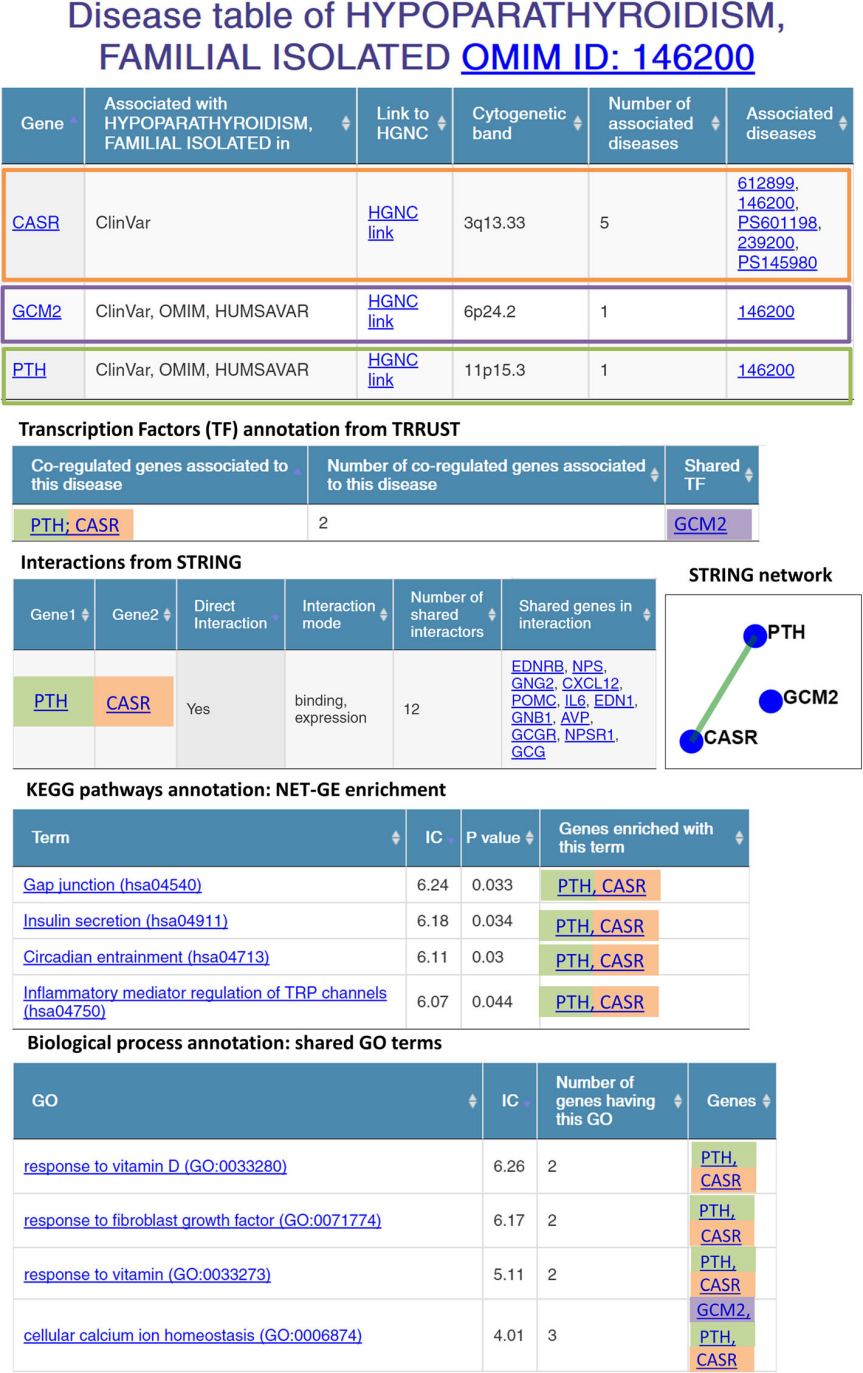
eDGAR page for hypoparathyroidism (OMIM 146200). In the figure, each gene is highlighted with a different color; the Transcription Factor annotation and the known interactions are reported, together with the simple graph describing them. A summary of the KEGG pathways enriched with NET-GE and the shared GO terms for BP is also provided

Two types of pages can be visualized: i) gene specific pages, reporting the associations to diseases and the available gene annotations; ii) disease specific pages, reporting the associations with genes and, in case of heterogeneous and polygenic diseases, the list of relationships linking the different genes, organized into different tables. Interactions from STRING, PDB, BIOGRID, CORUM, CENSUS can also be visualized by means of graphs, reporting direct and indirect interactions. The graphs show the gene associated with the disease as blue nodes and other genes in interactions as pale blue nodes; the direct interactions are visualized as green edges and the indirect interactions as thin black edges (see Fig. 3). Clicking on a node, the user is redirected to the correspondent gene page.

### A case study: Hypoparathyroidism

Hypoparathyroidism (OMIM 146200) is an endocrine deficiency disease characterized by low serum calcium levels, elevated serum phosphorus levels and absent or low levels of parathyroid hormone (PTH) in blood [35]. The metabolism of the patient may be altered: the vitamin D supply is inadequate and the magnesium metabolism is irregular. In some clinical panel, hypocalcemia can lead to dramatic effects such as tetany, seizures, altered mental status, refractory congestive heart failure, or stridor.

In eDGAR the familial isolated hypoparathyroidism (OMIM 146200) is associated with three different genes: GCM2 and PTH (both reported in OMIM, ClinVar and Humsavar) and CASR (reported only in ClinVar). CASR is an extracellular calcium-sensing receptor whose activity is mediated by G-proteins, PTH is the parathyroid hormone, whose function is to increase calcium level both by promoting the solution of bone salts and by preventing their renal excretion, and GCM2 (Glial cell missing homolog 2) is a probable transcriptional regulator, considering the SwissProt annotation. The “Transcription Factor (TF) annotation from TRRUST” table in eDGAR reports that GMC2 is a TF that regulates the expression of both PTH and CASR. Moreover, when considering “Interactions from STRING” table, PTH and CASR are in direct interaction, labelled as “binding” and “expression”. The shared BP GO terms with the highest IC values are “response to vitamin D” and “response to fibroblast growth factor”, both involving CASR and PTH. The response to vitamin D, whose metabolism is often altered in hypoparathyroidism, and a strict interplay between fibroblast growth factors and parathyroid hormone have been previously reported [36–38]. PTH and CASR are also involved in the same REACTOME pathways related to GPCR ligand binding and signaling. No shared KEGG term is found.

NET-GE enrichment for BP for the three genes include new terms endowed with high IC values, like “regulation of amino acid transport”, “negative regulation of muscle contraction”. Some of these new annotations are related to the severe symptoms of hypothyroidisms, namely tetany and seizure. NET-GE allows retrieving enriched KEGG pathways, such as “Circadian entrainment (hsa04713)”, “Inflammatory mediator regulation of TRP channels (hsa04750)”, “Gap junction (hsa04540)” and “Insulin secretion (hsa04911)”. None of the three genes is directly involved in the four pathways; PTH and CASR are part of the networks defined by NET-GE exploiting the STRING network. Interestingly, these new annotations highlight previously reported impairments of both circadian rhythms impairment and insulin secretion associated with hypoparathyroidism [39, 40].

Figure 3 reports a summary of the information provided by eDGAR for hypothyroidism (OMIM 146200), showing how it allows to collect the different types of relations among the involved genes in a unique page integrating data from many resources.

## Conclusions

eDGAR is a resource for the study of the associations between genes and diseases. It collects 2672 diseases, associated with 3658 different genes, for a total number of 5729 gene-disease associations. The novelty of eDGAR is the integration of different sources of gene annotation and in particular, for the 621 heterogeneous/polygenic diseases, eDGAR offers the possibility of analyzing functional and structural relations among co-involved genes. We provide direct interactions between pairs of genes (reported in STRING or BIOGRID) for 291 diseases and indirect interactions for some other 250 diseases. For 273 diseases, at least one pair of genes is under regulatory interaction of the same TF, while 39 disease are associated with genes being a TF/target couple. For 612 diseases, at least one pair of genes share GO terms and/or KEGG/REACTOME pathways. In particular, genes involved in the same disease most frequently share terms of the BP sub-ontology. This is confirmed also when analyzing the statistically significant functional terms enriched with NET-GE for 606 diseases. The relations among genes involved in the same disease are often complex and different pairs of genes are linked in different ways. eDGAR is a resource for better tackling the complexity of gene interactions at the basis of multigenic diseases. The database will be updated following the major releases of the different underlying data resources at least once a year.

## Additional file

Additional file 1: Figure S1. Distribution of median IC values of GO terms for genes involved in multigenic diseases. A: GO terms shared by genes; B: GO terms enriched with NET-GE. For each multigenic disease, IC value of gene-associated GO terms (of the three different roots) are evaluated (Eq. 1). In the figure the median IC for each disease is shown. The frequency is computed with respect to the total number of multigenic diseases (621). When IC = 0, genes associated with multigenic disease do not share or enrich GO terms (panel A and B respectively). (PNG 393 kb)
