# Supplementary figures and images for: eDGAR: a database of Disease-Gene Associations with annotated Relationships among genes
Source: BMC Genomics. 2017 Aug 11;18(Suppl 5):554. doi: 10.1186/s12864-017-3911-3 (PMC5558190; doi:10.1186/s12864-017-3911-3)

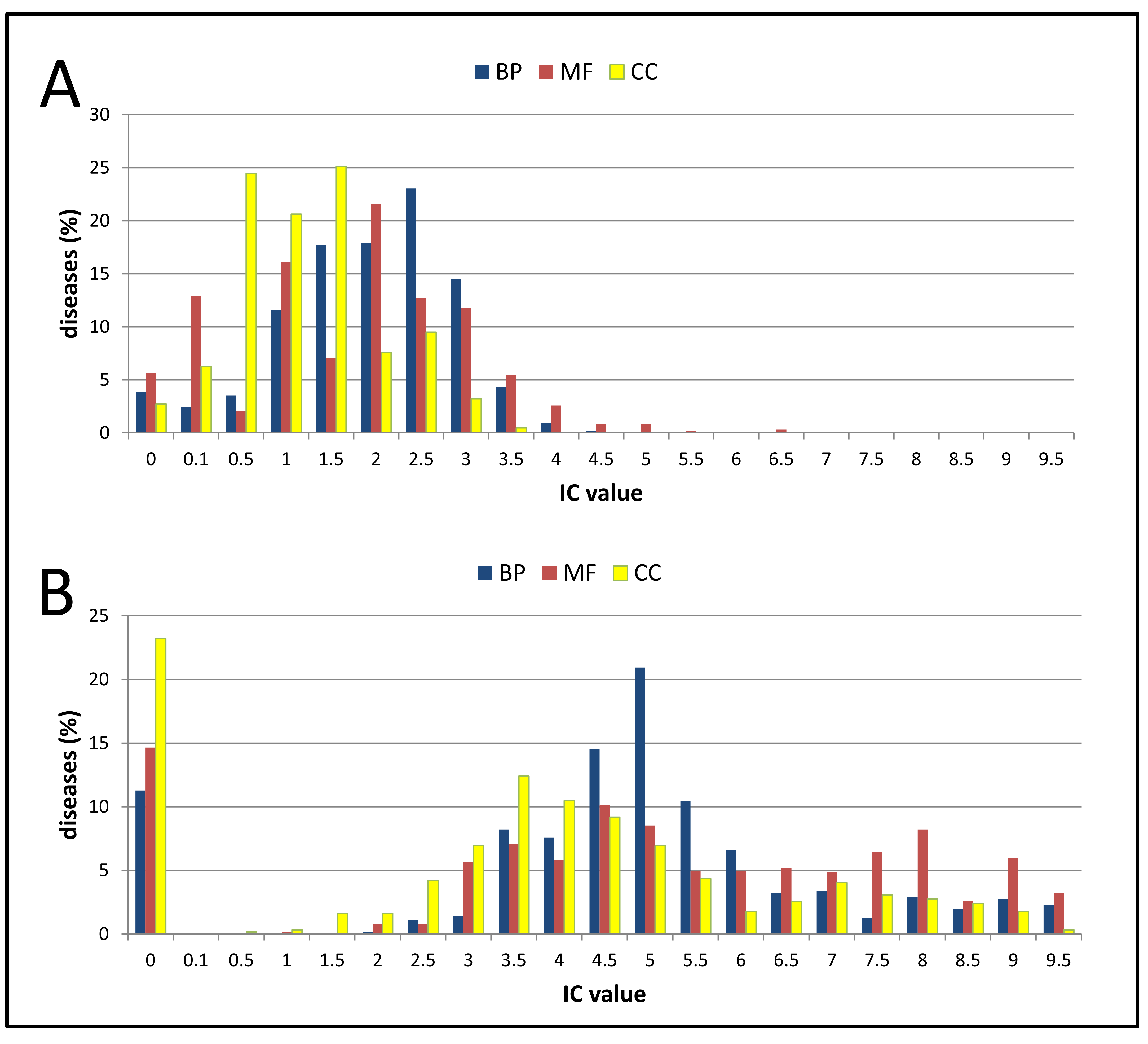

Supplement: Additional file 1: Figure S1. — Distribution of median IC values of GO terms for genes involved in multigenic diseases. A: GO terms shared by genes; B: GO terms enriched with NET-GE. For each multigenic disease, IC value of gene-associated GO terms (of the three different roots) are evaluated (Eq. 1). In the figure the median IC for each disease is shown. The frequency is computed with respect to the total number of multigenic diseases (621). When IC = 0, genes associated with multigenic disease do not share or enrich GO terms (panel A and B respectively). (PNG 393 kb) [file 12864_2017_3911_MOESM1_ESM.png]
